# Performance of Point-of-Care Ultrasonography in Confirming Feeding Tube Placement in Mechanically Ventilated Patients

**DOI:** 10.3390/diagnostics13162679

**Published:** 2023-08-15

**Authors:** Thanalachumy Ragunathan, Rufinah Teo, Aliza Mohamad Yusof, Siti Nidzwani Mohamad Mahdi, Azarinah Izaham, Chian Yong Liu, Maryam Budiman, Syarifah Noor Nazihah Sayed Masri, Raha Abdul Rahman

**Affiliations:** Department of Anaesthesiology & Intensive Care, Faculty of Medicine, Universiti Kebangsaan Malaysia, Jalan Yaacob Latif, Bandar Tun Razak, Kuala Lumpur 56000, Malaysia; thana122@yahoo.com (T.R.); alizamyusof@gmail.com (A.M.Y.); nidzwani@ppukm.ukm.edu.my (S.N.M.M.); azaizaham@ppukm.ukm.edu.my (A.I.); chianyong@yahoo.com (C.Y.L.); dr.maybud@gmail.com (M.B.); syarifahnoornazihah@gmail.com (S.N.N.S.M.); raha@ppukm.ukm.edu.my (R.A.R.)

**Keywords:** feeding tube, ultrasonography, intensive care units, point of care, sensitivity and specificity

## Abstract

Background: A feeding tube (FT) is routinely placed in critically ill patients, and its correct placement is confirmed with a chest X-ray (CXR), which is considered the gold standard. This study evaluated the diagnostic accuracy of ultrasonography (USG) in verifying FT placement compared to a CXR in an intensive care unit (ICU). Method: This was a prospective single-blind study conducted on patients admitted to the ICU of a tertiary hospital in Malaysia. The FT placements were verified through a fogging test and USG at the neck and subxiphoid points. The results of confirmation of FT placement through USG were compared with those obtained using CXRs. Results: A total of 80 patients were included in this study. The FT positions were accurately confirmed by overall USG assessments in 71 patients. The percentage of FT placements correctly identified by neck USG was 97.5%, while the percentage of those identified by epigastric USG was 75%. The corresponding patients’ CXRs confirmed correct FT placement in 76 patients. The overall USG assessment had a sensitivity of 92.11% and specificity of 75%, a positive predictive value of 98.59%, and a negative predictive value of 33.33%. The USG findings also showed a significant association between FT size and BMI. FTs with a size of 14Fr were better visualized (*p* = 0.008), and negative USG findings had a significantly higher BMI (*p* < 0.001). Conclusion: USG is a simple, safe, and reliable bedside assessment that offers relatively high sensitivity in confirming correct FT placement in critically ill patients.

## 1. Introduction

In an intensive care unit (ICU), feeding tubes (FTs) are frequently inserted for critically ill patients and for those who are intubated and require ventilator support. Although the incidence of tube displacement is low (0.5–1.5%), serious complications have been reported because of tube displacement, including misplacement of the tube in the tracheobronchial tree, pneumothorax, pneumomediastinum, pneumonia, bronchopleural fistula, esophageal perforation, bleeding, aspiration, and even death [1,2].

Several methods have been extensively investigated for verifying FT placement, such as the use of a pH meter, aspiration of gastric content, auscultation of air, capnography, and radiographic confirmation. Although a pH value of 1–5.5 for aspirate can be used safely as the first-line exclusion of a misplaced FT in the respiratory tract, studies have found false-negative pH among patients who were undergoing acid reduction therapy [3,4]. Meta-analysis and review studies reported that capnography alone unreliably reflects FT placement and X-ray was unavoidable [5,6]. Therefore, the radiographic confirmation of FT placement is considered the gold standard [7,8]. However, the use of this method in ICU patients has several disadvantages, including radiation exposure, high cost, and extensive labor, and it is only applicable during the time frame when the radiographic examination is conducted [9].

Auscultation, which is considered a low-value tradition-based practice, is still widely used today by healthcare practitioners in assessing FT placement [10]. This is despite several recent publications that have called for an urgent deimplementation of this method, which is not supported by evidence-based clinical practice guidelines [10,11]. The lack of valid bedside methods for verifying FT placements serves as a major barrier to moving away from this practice, which has been described as inaccurate and may have a tendency of producing false-positive results if not properly implemented [11,12,13,14,15]. As such, an evidence-based alternative that is accurate and time-saving must be introduced to ensure the safety of patients.

In recent years, ultrasonography (USG) has gained popularity as an assessment tool to guide diagnosis and timely management in different critical care units, including emergency departments, ICUs, and high-dependency units, even though it is an operator-dependent skill and requires training and experience for accurate image interpretation [16]. USG allows immediate and non-invasive bedside evaluation in critical settings in which time is a limitation. It is commonly used for the placement of central venous catheters, assessment of hemodynamic status and cardiac function, and diagnosis of pneumothorax or pleural effusion. This method allows for real-time images of the FT’s passage to be obtained [17], while being able to verify the tube placement in the esophagus as well as the stomach through the epigastrium [18]. Outside of critical care settings, FT placement is frequently seen in community settings where adults are unable to achieve the daily nutritional requirement orally. These patients depend on the correct placement of FT by community nurses not just for nutrition but also for therapeutic purposes [19]. Transporting bedbound patients to an X-ray facility may incur additional cost, stress, and unnecessary irradiation, and USG may play a new and yet important role in catering to the needs of these patients.

Newer-generation USG models also provide repetitive evaluations with high-resolution dynamic images. This has resulted in the establishment of standard protocols for the evaluation of critically ill patients [20,21]. However, despite the numerous applications of USG, its use for FT placement verification in ICU and emergency department patients has not been well established [4,17]. The few studies conducted on this topic have emphasized USG as a promising method for FT placement verification, as the results have shown high sensitivity (97.5%) and specificity (99%) [22]. This study aimed to evaluate the performance of USG in verifying the correct placement of FTs in critically ill patients. The aim of USG examination was to visualize the FTs’ placement at the neck and epigastric points.

## 2. Materials and Methods

This prospective, single-blind operator study was conducted over 12 months (December 2020 to November 2021) in a tertiary university teaching hospital in Malaysia. Approval from the Universiti Kebangsaan Malaysia Research and Ethics Committee was obtained before the start of the study (approval code: JEP-2020-552) and was registered in clinical trial registry (Clinicaltrial ID NCT05307900).

The inclusion criteria for the present study were patients admitted to the ICU in the study center throughout the study period, requiring placement of an FT placement, and above the age of 18 years. Patients with any contraindications for FT insertion (e.g., coagulopathy or esophageal varices); history of post-gastric bypass surgeries; known history of nasopharyngeal, esophagus, or stomach carcinoma; neck trauma/swelling, including goiter; open wounds in the neck or epigastric region; and pregnancy were excluded. Written consent was obtained from the patient’s next of kin prior to recruitment into the study.

**Figure 1:**
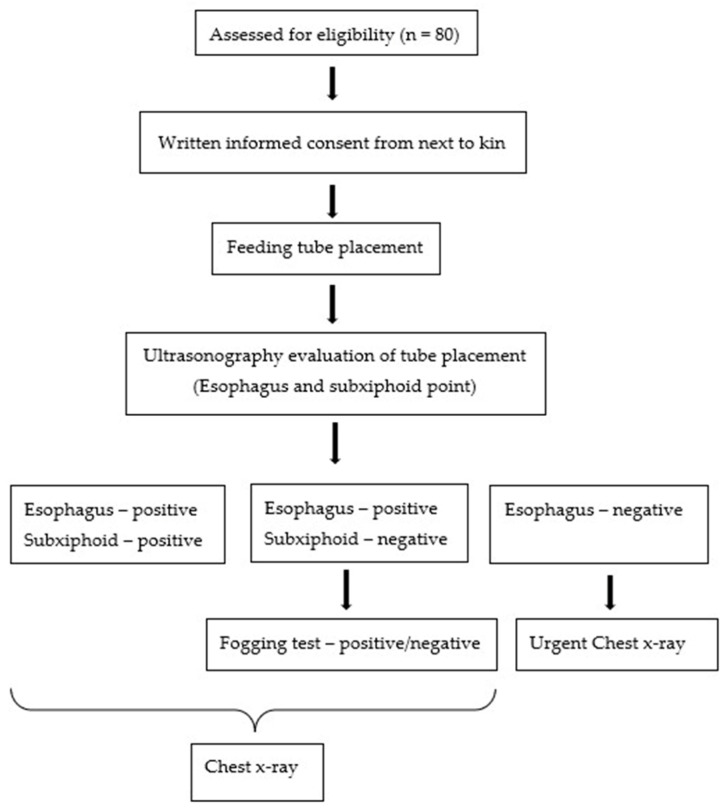
USG assessment methodology chart.

Figure 1 illustrates the systematic ultrasound protocol for positioning FT in patients that was used in the present study.

Polyvinylchloride (PVC) FTs with radio-opaque lines were inserted by anesthesiology trainees using the nose–ear–xiphoid method, which is used to measure the tube insertion distance by taking the sum of the distance from the tip of the patient’s nose to the earlobe and from the earlobe to the xiphoid. The tip of the tube was lubricated prior to insertion in all cases.

The FT was secured to the patient’s nose or cheek with adhesive tape once the clinical indicators of correct placement were obtained by either auscultation or aspiration of gastric content. Tube placement through USG was verified by an investigator trained by a radiologist in esophageal and gastric ultrasound. A portable ultrasound unit, “Sonosite SII” (Fujifilm Sonosite, Inc., Bothell, WA, USA), equipped with a linear probe (L38xi, 10–5 MHz) and a curved probe (C35x, 8–3 MHz) was used in this study.

**Figure 2:**
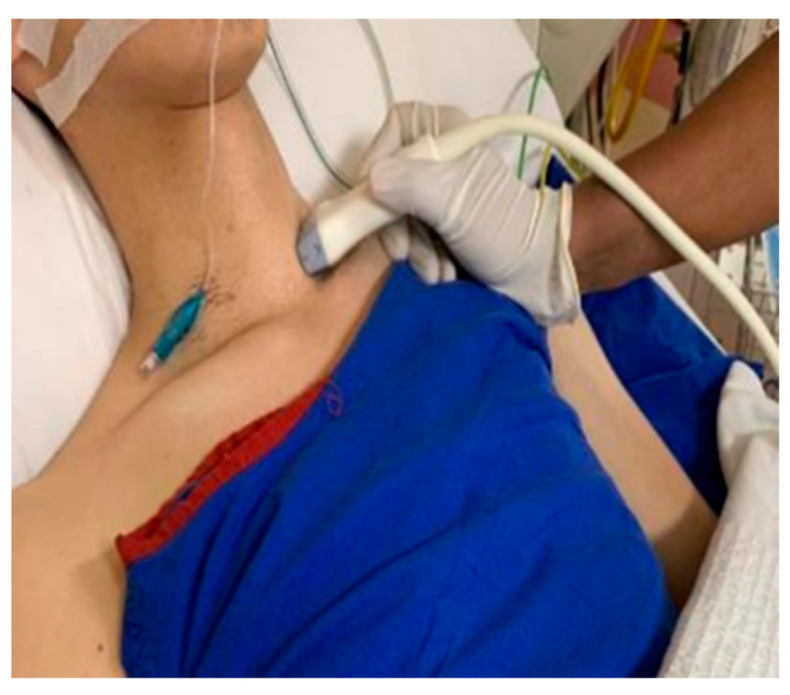
Placement of the ultrasound probe at the neck point.

**Figure 3:**
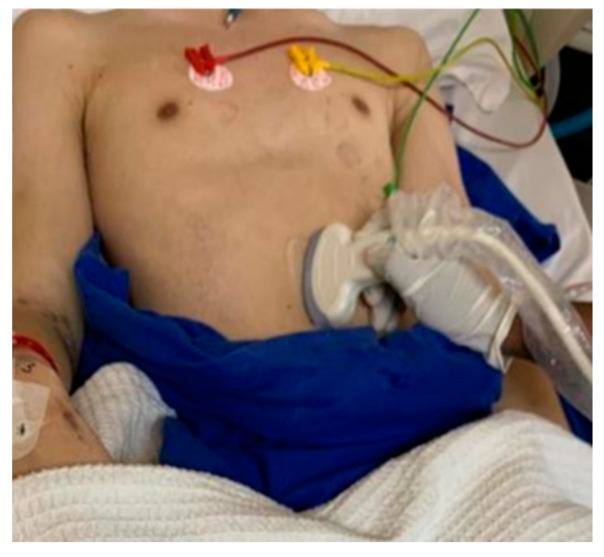
Placement of the ultrasound probe at the subxiphoid point.

The investigator performed a standardized technique in which the linear probe was placed transversely at the anterior neck and focused on the visible part of the esophagus (Figure 2). Then, to visualize the stomach, a curved probe was placed at the subxiphoid area (Figure 3), orientated toward the left upper abdominal quadrant, and angulated toward the left subcostal area. Subsequently, the gastric body was identified in the transverse plane adjacent to the left lobe of the liver, which was used as an internal landmark.

The USG examination was considered positive if the FT was visualized as a hyperechogenic circle posterior to the left thyroid lobe adjacent to the trachea and as a hyperechogenic point in the stomach. If the FT was detected in the esophagus and not in the stomach, 20 mL of air was injected through the FT using a pine-tip syringe while observing dynamic fogging in the stomach through ultrasound. The FT was considered to be in the gastric body based on the presence of fogging.

Chest X-rays (CXR) of all patients were taken post-ultrasound confirmation as the gold standard for confirming FT placement.

## 3. Statistical Analysis

The sample size estimation was adopted from Hajian-Tilaki [23], and the pre-determined value of specificity (0.957) was obtained from Yıldırım, Coşkun, Gökhan, Pamukçu Günaydın, Özhasenekler, and Özkula [22]. For an alpha error of 5%, the values were set at 1.96. The maximum marginal error of estimate (0.05) was pre-determined through the investigators’ clinical judgment. Anticipating a 20% dropout rate, the target sample size was set at 77 in this study.

All data analyses were performed using SPSS for Windows version 23.0 (IBM Corp., Armonk, NY, USA). For descriptive statistics, both mean and standard deviations were used to summarize the continuous variables after verifying the data distribution. Nominal variables were reported in terms of frequency and percentage. Inferential statistics, including the calculation of sensitivity, specificity, positive predictive values (PPVs), and negative predictive values (NPVs), were used to assess the diagnostic ability of the interventions with a confidence interval (CI) of 95%. The following formulas were used for the calculation of these parameters:

$$\mathrm{Sensitivity}=\frac{\mathrm{TP}}{\left(\mathrm{TP}+\mathrm{FN}\right)}\;\mathrm{Specificity}=\frac{\mathrm{TN}}{\left(\mathrm{TN}+\mathrm{FP}\right)}\;\mathrm{PPV}=\frac{\mathrm{TP}}{\left(\mathrm{TP}+\mathrm{FP}\right)}\;\mathrm{NPV}=\frac{\mathrm{TN}}{\left(\mathrm{TN}+\mathrm{FN}\right)}$$

where TP = true positive; TN = true negative; FP = false positive; and FN = false negative.

Fisher’s exact test was conducted to determine the association between FT size and USG findings. Additionally, the difference in BMI between patients with positive and negative USG was analyzed using an independent *t*-test. A *p*-value <0.05 was considered statistically significant.

## 4. Results

Eighty patients were included in this study, whose demographic and clinical characteristics are summarized in Table 1 and Table 2, respectively.

**Figure 4:**
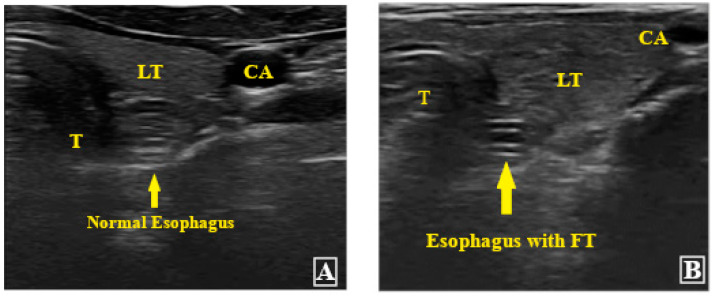
Neck point ultrasonography image (CA = carotid artery, LT = left thyroid lobe, T = trachea). The normal esophagus without the feeding tube (FT) (**A**) vs. with the FT (**B**).

**Figure 5:**
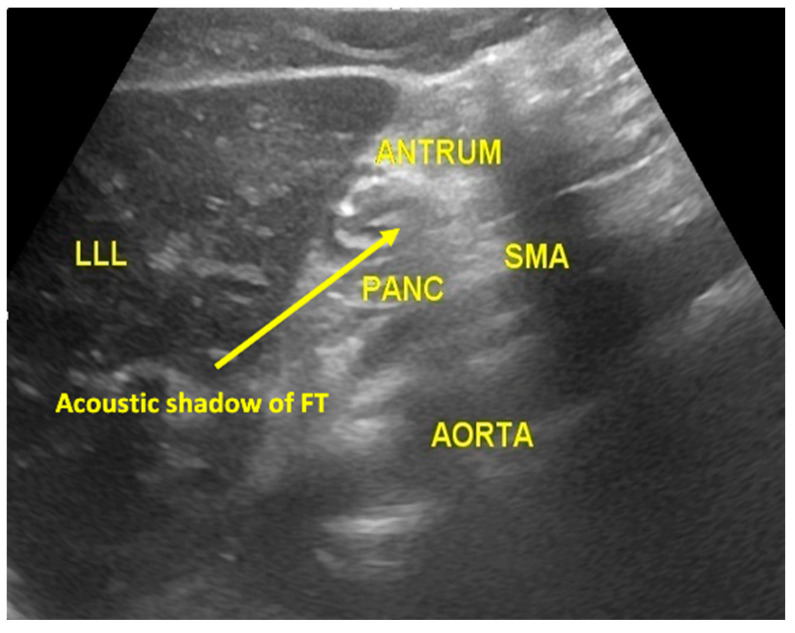
Subxiphoid area ultrasonography image (LLL = left liver lobe, SMA = small mesenteric artery, PANC = pancreas, ANTRUM = antrum of the stomach, AORTA = abdominal aorta).

**Figure 6:**
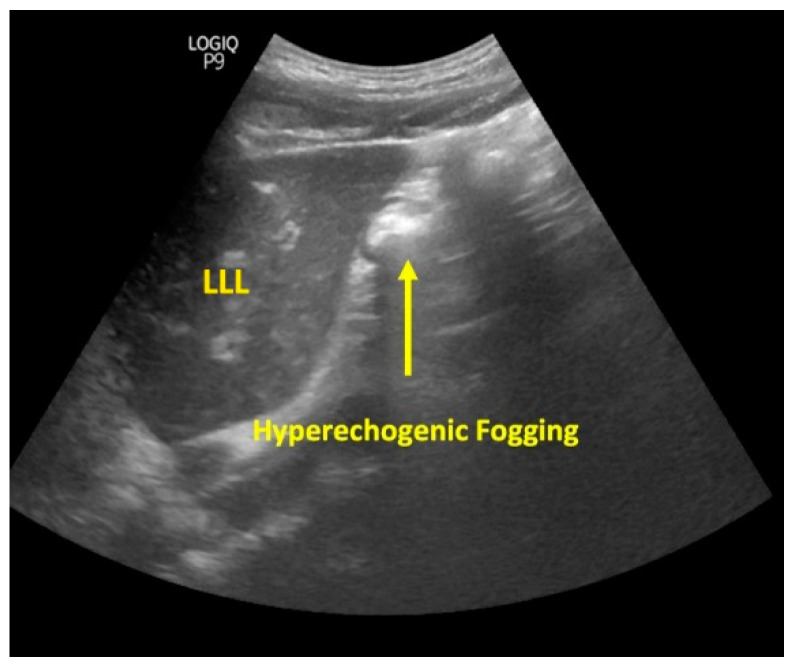
Fogging test ultrasonography image (LLL = left liver lobe).

Out of the 80 patients, 78 patients’ FTs could be visualized in the esophagus during the USG assessment (Figure 4). The remaining two patients had overall negative USG. The 78 patients were further assessed at the subxiphoid point, which could confirm correct FT placement in only 60 patients (Figure 5). The fogging test was positive in 11 out of the 18 patients with negative subxiphoid results (Figure 6). Therefore, the USG assessment could confirm correct FT placement in 71 patients compared to the gold standard CXR, which could confirm the same in 76 patients. Figure 4, Figure 5 and Figure 6 are representative ultrasound images obtained from the present study.

At the esophagus USG point, the highest sensitivity shown was 98.68%, which indicated that the FT was not displaced in the trachea (Table 3). Although the subxiphoid point showed a sensitivity of 77.63%, it had a specificity of 75%. However, when combined with the fogging test, the overall sensitivity and specificity values of the ultrasonography assessment (neck, subxiphoid, and fogging) were significantly higher at 92.11% and 75%, respectively.

**Figure 7:**
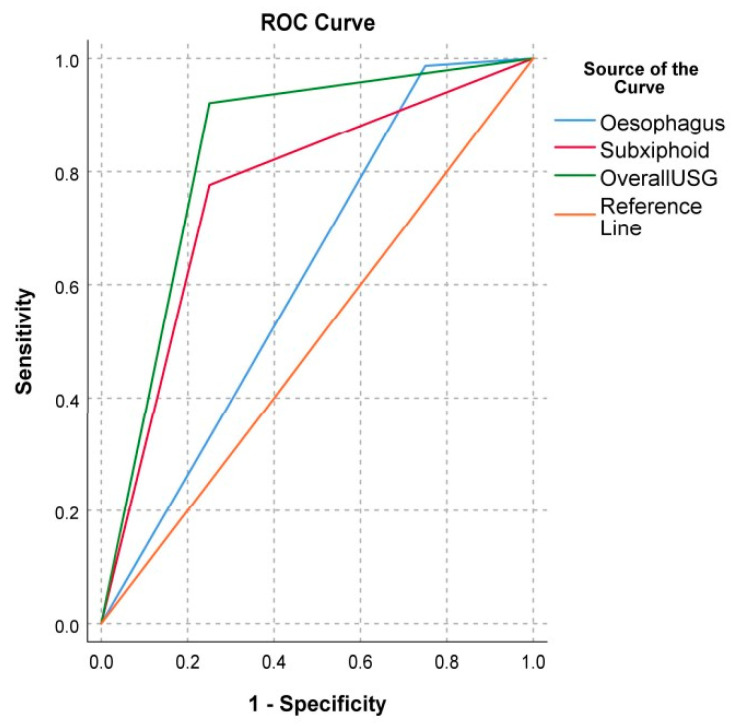
Receiver operating curve (ROC).

Upon converting the sensitivity and specificity percentages of each USG point in Table 3 to decimals, a receiver operating characteristic (ROC) curve was plotted. This yielded good area-under-the-curve results: 0.618 for the esophagus point, 0.763 for the subxiphoid point, and 0.836 for the overall USG (Figure 7).

Positive USG findings were significant at a lower BMI and 14 Fr FT. The visibility of the 14 Fr FT was as high as 93% (Table 4).

## 5. Discussion

The overall USG assessment in the present study had a sensitivity value of 92.11% (95% CI: 86.20, 98.01), specificity value of 75% (95% CI: 65.51, 84.49), PPV of 98.59% (95% CI: 96.01, 100.00), and NPV of 33.33% (95% CI: 23.00, 43.66. Correct FT placement at the neck point and subxiphoid point using the two-point USG technique was obtained in 71 out of 80 patients (88.75%). The high sensitivity of 92.11% with a ROC of 0.86 was comparable to the results obtained by previous studies. Ye et al. [24] developed a protocol for USG confirmation of nasointestinal FT placement recently. They carried out a single-center study that included 157 critically ill patients and achieved sensitivity and specificity values of 96.4% and 90%, respectively [24]. However, the success of this study required detailed knowledge of USG and the anatomy of intra-abdominal structures in order to identify correct nasointestinal tube positioning. In one of the earliest studies by Vigneau, the highest sensitivity value obtained was 98% [25]. This result was attributed to the use of metal nose FTs, which provided enhanced hyperechoic information and improved visibility of the ultrasound images. On the other hand, the present study used FTs made from PVC with radio-opaque lines and demonstrated a comparable result of 92.11%. Two subsequent smaller-sample-size studies by Gok, Kilicaslan, and Yosunkaya [17] and Dağlı et al. [26], which used PVC FTs, also achieved a good sensitivity result of 97% [17,26]. A comparative summary of the overall sensitivity and specificity rate between the present and past studies that utilized USG in FT detection can be found in Table 5.

There were two negative neck USG findings. One finding was a true negative, as the FT was coiled in the oral cavity, and the second was a false negative, which was secondary to suboptimal imaging, as the patient had class III obesity with a BMI of 42. The sensitivity of esophageal USG was 98.68%. Yıldırım, Coşkun, Gökhan, Pamukçu Günaydın, Özhasenekler, and Özkula [22] found a neck USG sensitivity that was slightly lower, at 91.5%, than both our study and that of Gok, Kilicaslan, and Yosunkaya [17] (98.68% and 98%, respectively). In their study, both negative neck findings were attributed to the coiling of FT in the oral cavity, which is common [17,22]. The sensitivity of neck USG is generally high because neck structure identification is much easier due to its superficial location. Even though the specificity was suboptimal, it was useful in eliminating the possibility of FT malposition in the airway. Thus, a second assessment at the subxiphoid point was required to confirm the location of the FT tip in the gastric body. Twenty patients had negative USG results at the subxiphoid point. We had one false-positive USG finding at the subxiphoid point due to the presence of a Tenckhoff catheter. The visibility of the FT in the gastric body is compromised by a few factors, such as full stomach, gas interposition, and the presence of other indwelling abdominal catheters. A dynamic fogging test improved the confirmation of placement, which was also demonstrated in Chenaitia et al. [27] and Yıldırım, Coşkun, Gökhan, Pamukçu Günaydın, Özhasenekler, and Özkula’s [22] studies [22,27].

There were instances of false positives in the present study when using the USG techniques. Negative findings were obtained from the CXR evaluation, which is considered the gold standard practice, for these cases. These instances could be attributed to a few factors, which include malposition due to movement that may have occurred in the space of time between when the USG and CXR were performed. Moreover, as the USG is a highly operator-dependent technique that requires sufficient skill and training [28], misinterpretation by the operator as well as artifacts may have resulted in the false-positive findings. Unsatisfactory image captures due to patient positioning, tube visibility, or image resolution as well as operator misinterpretation may have resulted in compromised quality of the CXR findings, yielding a negative result. To avoid situations of false results as well as improve safety and efficiency, the usage of more than one confirmatory test is encouraged [29].

Recently, point-of-care USG has gained popularity as a rapid diagnostic and treatment tool in healthcare settings, especially in critical care, where decision-making time is crucial. The biggest advantages of ultrasonography are that it can be performed swiftly and instantaneously at the bedside and used repetitively without any risk of radiation exposure. Most importantly, the images can be viewed in real-time, in contrast to X-ray examinations. Despite these advantages, there are only a few published studies on USG confirmation of FT placement in ICU patients with highly positive results.

USG assessment requires less time owing to its widespread availability in most critical care units. Vigneau, Baudel, Guidet, Offenstadt, and Maury [25] and Acosta Pedemonte et al. [30] found that confirming FT placement with USG took significantly less time than confirming it with CXRs (10 min vs.180 min) [25,30]. In our study, the mean time taken for USG assessment was 15.50 ± 2.43 min. This was mainly because the assessment was performed at two different points, in contrast to other studies that performed their assessments at a single point. Nonetheless, the time taken for FT placement confirmation through USG is still far less than that taken for confirmation through CXRs.

We consider the FT sizes used in this study as our limitation. There were only two sizes used: 12 Fr (n = 9) and 14 Fr (n = 71). Four of the nine patients with a 12 Fr FT showed a false-negative result. There was a significant association between FT size and ultrasound visibility: a larger FT conferred better visibility in ultrasonography (*p* = 0.008). The limitation of our study is that the sample size for the 12 Fr FT was small, which could have contributed to this finding. Data comparing FT sizes and their relation to ultrasonography visibilities are scarce. To our knowledge, no studies have focused on the relationship between FT size and ultrasound outcomes. Yıldırım, Coşkun, Gökhan, Pamukçu Günaydın, Özhasenekler, and Özkula [22] fixed the FT size to 16 Fr and obtained 95.74% sensitivity, which was slightly higher than that obtained in our study [22]. Additionally, Chenaitia, Brun, Querellou, Leyral, Bessereau, Aimé, Bouaziz, Georges, and Louis [27] used various FT sizes of 18 Fr, 16 Fr, and 14 Fr and obtained sensitivity values of 100%, 98%, and 81%, respectively [27].

The second limitation of this study was the lack of obese patients specifically recruited as the cohort. Although they were not excluded, patients with negative USG findings had a significantly higher BMI (32.33 ± 6.80 kg/m^2^). There are only a few studies that have included BMI correlation in their analysis, including that of Nedel et al. [31] where BMI (ranging from 19.2 to 28 kg/m^2^) patients were recruited and a sensitivity of 97% was achieved [31]. However, no data correlation was made regarding FT size or BMI. Although Komagata et al. [32] commented on the correlation between BMI and the negative results obtained, they did not include the corresponding data in their study [32].

## 6. Conclusions

Two-point USG is a simple bedside examination for FT confirmation with a high sensitivity of 92.11%, which is comparable to that obtained using X-ray imaging. A lower BMI and larger FT size provide better USG image visibility.

## Figures and Tables

**Table 1:** Demographic characteristics of the patients.

Variables		Mean ± SD
Age (years)		52.95 ± 18.28
Weight (kg)		69.41 ± 14.50
Height (cm)		163.45 ± 6.62
BMI (kg/m^2^)		25.99 ± 4.81
**Variables**		**n (%)**
Gender	Male Female	50 (62.5) 30 (37.5)
Race	Malay Chinese Indian Others	39 (48.8) 24 (30.0) 4 (17.5) 3 (3.8)
Diabetes mellitus Hypertension		35 (43.8) 21 (26.3)
Renal disease		11 (13.8)
IHD		13 (16.3)
Others (bronchial asthma, cancer, etc.)		20 (25.0)

**Table 2:** Clinical characteristics of the patients.

Variables		n (%)
Primary Discipline	Medical Surgery	53 (66.3) 27 (33.7)
Size (Fr)	12 14	9 (11.3) 71 (88.8)
**Ultrasonography Outcomes**		
Placement in esophagus	Yes No	78 (97.5) 2 (2.5)
Placement in subxiphoid	Yes No	60 (75.0) 18 (25.0)
Presence of fogging	Yes No	11 (61.1) 7 (38.9)
Overall ultrasonography findings	Positive Negative	71 (88.7) 9 (11.3)
Duration of ultrasound assessment in minutes, mean ± SD	15.50 ± 2.43	
**Chest X-ray findings**	Yes No	76 (95.0) 4 (5.0)

**Table 3:** Sensitivity and specificity for each ultrasonography point.

USG	Chest X-ray * (n)		Sensitivity (%)	Specificity (%)	PPV (%)	NPV (%)
	Negative	Positive				
**Esophagus**			98.68 (96.19, 100.00)	25.00 (15.51, 34.49)	96.15 (91.94, 100.00)	50.00% (39.04, 60.96)
- Negative	1	1				
- Positive	3	75				
**Subxiphoid**			77.63 (68.50, 86.76)	75.00 (65.51, 84.49)	98.33 (95.53, 100.00)	15.00 (7.18, 22.82)
- Negative	3	17				
- Positive	1	59				
**Overall** ^#^			92.11 (86.20, 98.01)	75.00 (65.51, 84.49)	98.59 (96.01, 100.00)	33.33 (23.00, 43.66)
- Negative	3	6				
- Positive	1	70				

Values are expressed in number (95% confident interval). * Indicating Chest X-ray result of correct or incorrect FT tube placement. ^#^ Subxiphoid visualization of FT and fogging test.

**Table 4:** Association between BMI and FT size and USG findings.

	Overall USG		Mean Difference	*p*-Value
	Positive, n (%)	Negative, n (%)		
Gastric tube size (Fr)				
12	5 (7.0)	4 (44.4)	-	0.008 ^a,^*
14	66 (93.0)	5 (55.6)		
Mean BMI (Kg/m^2^)	24.96 ± 3.67	34.10 ± 5.27	−7.24 (95% CI: −12.15, −2.33)	<0.001 ^b,^*

^a^ Fisher’s exact test, ^b^ independent sample *t*-test. * Statistically significant (*p* < 0.05).

**Table 5:** Comparison of overall sensitivity and specificity of various studies utilizing USG.

Study	Sensitivity Rate (%)	Specificity Rate (%)	Number of Patients (n)	Remarks
Present study	92.1	75.0	80	
Ye, Cheng, Chai, Peng, Liu, and Jing [24]	96.4	90.0	157	Studied nasointestinal tube placement with USG
Vigneau, Baudel, Guidet, Offenstadt, and Maury [25]	97	-	35 procedures in 33 patients	Usage of metal nose FT
Gok, Kilicaslan, and Yosunkaya [17]	93		56	-
Dağlı, Bayır, Dadalı, Tokmak, and Erbesler [26]	97	-	34	-
Yıldırım, Coşkun, Gökhan, Pamukçu Günaydın, Özhasenekler, and Özkula [22]	95.7	100	49	-

## Data Availability

The data presented in this study are available on request from the corresponding author.

## References

[1] Bankier A.A., Wiesmayr M.N., Henk C., Turetschek K., Winkelbauer F., Mallek R., Fleischmann D., Janata K., Herold C.J. (1997). Radiographic detection of intrabronchial malpositions of nasogastric tubes and subsequent complications in intensive care unit patients. Intensiv. Care Med..

[2] Harris M.R.R.M., Huseby J.S. (1989). Pulmonary complications from nasoenteral feeding tube insertion in an intensive care unit: Incidence and prevention. Crit. Care Med..

[3] Boeykens K., Steeman E., Duysburgh I. (2014). Reliability of pH measurement and the auscultatory method to confirm the position of a nasogastric tube. Int. J. Nurs. Stud..

[4] Kim H.M., So B.H., Jeong W.J., Choi S.M., Park K.N. (2012). The effectiveness of ultrasonography in verifying the placement of a nasogastric tube in patients with low consciousness at an emergency center. Scand. J. Trauma Resusc. Emerg. Med..

[5] Chau J.P., Lo S.H., Thompson D.R., Fernandez R., Griffiths R. (2011). Use of end-tidal carbon dioxide detection to determine correct placement of nasogastric tube: A meta-analysis. Int. J. Nurs. Stud..

[6] Ryu J.-A., Choi K., Yang J.H., Lee D.-S., Suh G.Y., Jeon K., Cho J., Chung C.R., Sohn I., Kim K. (2016). Clinical usefulness of capnographic monitoring when inserting a feeding tube in critically ill patients: Retrospective cohort study. BMC Anesthesiol..

[7] Metheny N.A., Stewart B.J., Smith L., Yan H., Diebold M., Clouse R.E. (1999). pH and Concentration of Bilirubin in Feeding Tube Aspirates As Predictors of Tube Placement. Nurs. Res..

[8] Taylor S.J. (2013). Confirming nasogastric feeding tube position versus the need to feed. Intensiv. Crit. Care Nurs..

[9] Snaith B., Flintham K. (2015). Radiology responsibilities post NPSA guidelines for nasogastric tube insertion: A single centre review. Radiography.

[10] Bourgault A.M., Upvall M.J., Nicastro S., Powers J. (2022). Challenges of de-implementing feeding tube auscultation: A qualitative study. Int. J. Nurs. Pract..

[11] Bourgault A.M.P., Powers J.P., Aguirre L.D., Hines R.B., Sebastian A.T.M., Upvall M.J.P. (2020). National Survey of Feeding Tube Verification Practices: An Urgent Call for Auscultation Deimplementation. Dimens. Crit. Care Nurs..

[12] Fan E.M.P., Tan S.B., Ang S.Y. (2017). Nasogastric tube placement confirmation: Where we are and where we should be heading. Proc. Singap. Healthc..

[13] Hsieh S.-W., Chen H.-S., Chen Y.-T., Hung K.-C. (2017). To characterize the incidence of airway misplacement of nasogastric tubes in anesthetized intubated patients by using a manometer technique. J. Clin. Monit. Comput..

[14] Neumann M.J., Meyer C.T., Dutton J.L., Smith R. (1995). Hold that x-ray: Aspirate pH and auscultation prove enteral tube place-ment. J. Clin. Gastroenterol..

[15] Turgay A.S., Khorshid L. (2010). Effectiveness of the auscultatory and pH methods in predicting feeding tube placement. J. Clin. Nurs..

[16] Nguyen L., Lewiss R.E., Drew J., Saul T. (2012). A novel approach to confirming nasogastric tube placement in the ED. Am. J. Emerg. Med..

[17] Gok F., Kilicaslan A., Yosunkaya A. (2015). Ultrasound-Guided Nasogastric Feeding Tube Placement in Critical Care Patients. Nutr. Clin. Pract..

[18] Petitpas F., Kerforne T., Lacroix C., Mimoz O. (2012). Comment on “A novel approach to confirming nasogastric tube placement in the ED”. Am. J. Emerg. Med..

[19] Mak M.Y., Tam G. (2020). Ultrasonography for nasogastric tube placement verification: An additional reference. Br. J. Community Nurs..

[20] Kirkpatrick A.W., Sirois M., Laupland K.B., Liu D., Rowan K., Ball C.G., Hameed S.M., Brown R., Simons R., Dulchavsky S.A. (2004). Hand-Held Thoracic Sonography for Detecting Post-Traumatic Pneumothoraces: The Extended Focused Assessment With Sonography For Trauma (EFAST). J. Trauma Acute Care Surg.

[21] Lichtenstein D.A. (2015). BLUE-protocol and FALLS-protocol: Two applications of lung ultrasound in the critically ill. Chest.

[22] Yıldırım Ç., Coşkun S., Gökhan Ş., Pamukçu Günaydın G., Özhasenekler A., Özkula U. (2018). Verifying the Placement of Nasogastric Tubes at an Emergency Center: Comparison of Ultrasound with Chest Radiograph. Emerg. Med. Int..

[23] Hajian-Tilaki K. (2014). Sample size estimation in diagnostic test studies of biomedical informatics. J. Biomed. Inform..

[24] Ye R., Cheng X., Chai H., Peng C., Liu J., Jing J. (2021). A systemic ultrasound positioning protocol for nasointestinal tube in critically ill patients. Crit. Care.

[25] Vigneau C., Baudel J.-L., Guidet B., Offenstadt G., Maury E. (2005). Sonography as an alternative to radiography for nasogastric feeding tube location. Intensiv. Care Med..

[26] Dagli R., Bayir H., Dadali Y., Tokmak T.T., Erbesler Z.A. (2017). Role of Ultrasonography in Detecting the Localisation of the Nasoenteric Tube. Turk. J. Anesthesia Reanim..

[27] Chenaitia H., Brun P.-M., Querellou E., Leyral J., Bessereau J., Aimé C., Bouaziz R., Georges A., Louis F. (2012). Ultrasound to confirm gastric tube placement in prehospital management. Resuscitation.

[28] Atalay Y., Polat A., Ozkan E., Tomak L., Aygun C., Tobias J.D. (2019). Bedside ultrasonography for the confirmation of gastric tube placement in the neonate. Saudi J. Anaesth..

[29] Taylor S., Manara A.R. (2021). X-ray checks of NG tube position: A case for guided tube placement. Br. J. Radiol..

[30] Pedemonte N.A., Bagilet D., Rocchetti N., Torresan G., Rodríguez N., Settecase C. (2021). Color doppler ultrasound is a precise method to evaluate the position of the nasogastric tube in critical ill patients. Med. Intensiv..

[31] Nedel W.L., Jost M.N.F., Filho J.W.F. (2017). A simple and fast ultrasonographic method of detecting enteral feeding tube placement in mechanically ventilated, critically ill patients. J. Intensiv. Care.

[32] Komagata K., Yabunaka K., Nakagami G., Ikeda M., Takehara K., Takemura Y., Sanada H. (2018). Confirming the placement of nasogastric tubes by hand-carried ultrasonography device. J. Nurs. Sci. Eng..

